# iMKT: the integrative McDonald and Kreitman test

**DOI:** 10.1093/nar/gkz372

**Published:** 2019-05-13

**Authors:** Jesús Murga-Moreno, Marta Coronado-Zamora, Sergi Hervas, Sònia Casillas, Antonio Barbadilla

**Affiliations:** Institut de Biotecnologia i de Biomedicina and Departament de Genètica i de Microbiologia, Universitat Autònoma de Barcelona, 08193 Bellaterra, Barcelona, Spain

## Abstract

The McDonald and Kreitman test (MKT) is one of the most powerful and widely used methods to detect and quantify recurrent natural selection using DNA sequence data. Here we present iMKT (acronym for integrative McDonald and Kreitman test), a novel web-based service performing four distinct MKT types. It allows the detection and estimation of four different selection regimes −adaptive, neutral, strongly deleterious and weakly deleterious− acting on any genomic sequence. iMKT can analyze both user's own population genomic data and pre-loaded *Drosophila melanogaster* and human sequences of protein-coding genes obtained from the largest population genomic datasets to date. Advanced options in the website allow testing complex hypotheses such as the application example showed here: do genes located in high recombination regions undergo higher rates of adaptation? We aim that iMKT will become a reference site tool for the study of evolutionary adaptation in massive population genomics datasets, especially in Drosophila and humans. iMKT is a free resource online at https://imkt.uab.cat.

## INTRODUCTION

One of the most striking evidence of the power of natural selection is the characteristic footprints that it leaves on the patterns of genetic variation. A growing number of statistical methods to analyze genomic data allows us to detect and quantify adaptation and other selection regimes in the genome at different temporal scales (reviewed in 1).

The McDonald and Kreitman test (MKT, 2) is one of the most powerful and robust methods we have to detect the action of natural selection at the DNA level. MKT tests for the presence of recurrent positive (adaptive) selection on a gene or genome region. Unlike the *ω* = *d*_N_/*d*_S_ ratio (3), which uses only divergence data among species to compute the quotient of the number of non-synonymous (*d*_N_) to synonymous (*d*_S_) substitutions, the MKT uses both polymorphic and divergence data. Polymorphic data allows taking into account purifying selection on divergent non-synonymous sites, significantly increasing the detection power of recurrent positive selection. The MKT covers the evolutionary period spanning from the divergence of the outgroup species to the present. The null model of MKT is the neutral hypothesis (4,5). Because infrequent adaptive mutations fix fast relatively to common neutral mutations, they contribute almost exclusively to divergence and not to polymorphism; therefore, an excess of the divergence ratio relative to the polymorphism ratio is the signal of positive selection. The fraction of adaptive nonsynonymous substitutions (*α*) can be estimated from the MKT data (6,7).

The main drawback of MKT is that it assumes strict neutrality of segregating sites. Because weak negative selection abounds in the genomes (1), *α* estimates are biased downward. Several MKT methodological extensions try to correct the bias. In Appendix 1, four MKT approaches are listed: (i) the standard (original) MKT (2); (ii) the Fay, Wyckoff and Wu correction (_FWW_MKT) (8); (iii) the extended MKT (eMKT) (9) and (iv) the asymptotic MKT (aMKT) (10). Each method has pros and cons as discussed in Appendix 1, and for the comparison of their different outputs, it would be very convenient to have a web service to perform at once the four MKTs. Existing web servers compute either the standard MKT (11,12) or more recently the aMKT (13). None of them contains pre-loaded population genomics data of representative species as *Drosophila melanogaster* or humans.

**Figure 1.**
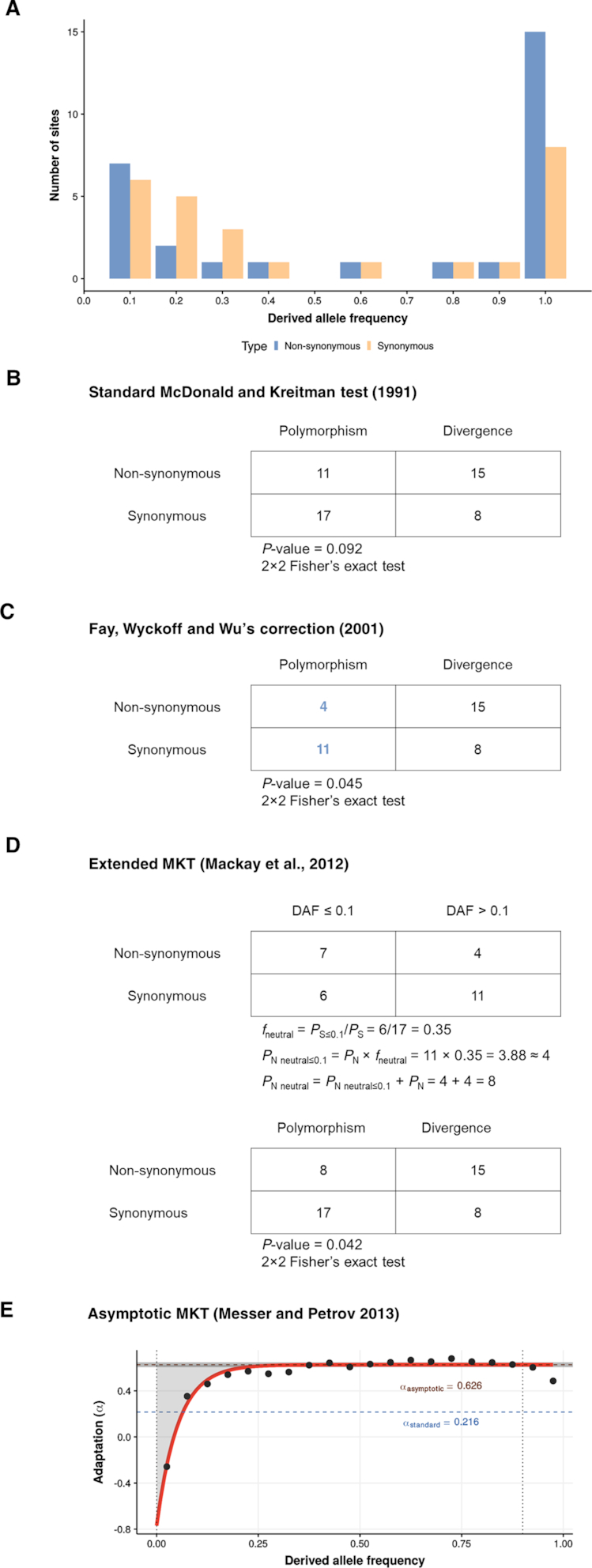
Comparison of the four MKT methods implemented in iMKT. (**A**) The hypothetical derived allele frequency (DAF) spectrum of synonymous and non-synonymous classes for a gene exhibiting an excess of both slightly deleterious and fixed non-synonymous differences with n = 10 sampled chromosomes. (**B**) The **standard MKT** for this gene (*P*-value = 0.09, 2 × 2 Fisher's exact test). (**C**) The 2 × 2 table by **Fay, Wyckoff and Wu's correction** (24) taking into account only polymorphism found on more than one chromosome (*P*-value = 0.045, 2 × 2 Fisher's exact test). (**D**) **Extended MKT** (9). The count of segregating sites in non-synonymous sites is partitioned into the number of neutral variants and the number of weakly deleterious variants. *P*_N_ is substituted with the number of nonsynonymous polymorphisms that is neutral (*P*-value = 0.042, 2 × 2 Fisher's exact test). (**E**) **Asymptotic MKT**. Example of the result of asymptotic MKT using *D. melanogaster* 2R chromosome and *D. simulans* as outgroup. The two vertical lines show the limits of the *x* cutoff interval used (in the example [0,0.9]). Black dots indicate the binned values for each DAF category. The solid red curve shows the fitted fit(*x*). The dashed red line is the final asymptote. The dark gray band indicates the 95% CI around the estimation. The blue dashed line shows the estimated using the standard MKT for comparison. For MKT methods definitions, see Appendix 1. Adapted and expanded from 29.

Here we present iMKT (acronym for integrative McDonald and Kreitman test), a web-based service performing the four MKT types described in Appendix 1 and Figure 1. It allows the detection and estimation of four selection regimes (adaptive, neutral, strongly deleterious and weakly deleterious) acting on protein-coding DNA sequences. The benefit of this tool is fourfold.

1. Four MKTs, two of which were not previously available as open software packages, can be performed at once to analyze user's own population genomic data in a simple interface offered by a web-based service.
2. It allows the simultaneous comparisons of the results of the different MKTs, which behave differently according to different properties of the data.
3. Taking advantage of the copious information gathered in previous population genome browsers, PopFly (14) and PopHuman (15), it offers a fast tool to estimate the different selective regimes on thousands of *D. melanogaster* and human protein-coding genes on several worldwide populations.
4. It allows comparing the selective regimes of a set of coding genes (selected according to the user's criterion, such as recombination rate bins or chromosome localization) with those of the genome-wide distribution in both humans and *D. melanogaster*.

The incessant accumulation of massive genome data makes this website a timely resource to describe and quantify natural selection for any biological species at the genome level.

## MATERIALS AND METHODS

### Input data

The iMKT server can analyze both user's own population data and pre-loaded data of *D. melanogaster* or human protein-coding genes.

In the first case, the user can upload as input either polymorphism and divergence data or aligned multi-FASTA files. For polymorphism and divergence data, the user must upload two files: (i) a tab-delimited file containing the distribution of Derived Allele Frequencies (DAF) (16) of all segregating (polymorphic) variants for two types of sites (putatively under selection and putatively neutral) and (ii) a file containing the counts of divergent positions for the two site types. For aligned multi-FASTA files, the user needs to enter one or more files containing aligned protein-coding sequences for at least two sequences of the same species to estimate polymorphism counts, and one orthologous sequence from an outgroup species to estimate divergence and infer ancestral alleles. Examples of such files are provided at the website.

For analyzing *D. melanogaster* or human protein-coding genes, the user can use the population genomic data available in the web server. In this case, the user can either submit a list of protein-coding genes or select them from the list provided, and select the population(s) and preferred method(s) to analyze the selective regimes on a group of protein-coding genes.

### Population genetics pipeline for *D. melanogaster* and human data

We have designed and implemented a custom pipeline for analyzing the Drosophila Genome Nexus (17,18) and Human 1000GP Phase III (19) data, which could potentially be escalated to any available genomic data source. The pipeline pre-calculates the DAF and number of divergent synonymous and nonsynonymous sites, which are needed to further perform on-the-fly MKTs. A total of 13 753 protein-coding genes for 16 *D. melanogaster* populations (17,18) and 20 643 protein-coding genes for 26 human populations of distinct geographical origin (19) were analyzed. Pre-calculated DAF and divergence values are stored in the server. The complete pipeline is available as a Jupyter Notebook at https://github.com/BGD-UAB/iMKTData to allow its reproducibility.

### Data retrieval

#### *D. melanogaster* population genomic data

Variation data generated by the Drosophila Genome Nexus, together with divergence data between *D. melanogaster* and *D. simulans*, was retrieved from PopFly (14) in FASTA format. Only populations with at least four genome sequences and less than 20% of missing or ambiguous nucleotides each (after filtering by identity by descent, admixture and heterozygosity) were included. DAF spectrum by functional classes was estimated by resampling a number of lines with nucleotide information (excluding undetermined sites, *N* bases) at each position without replacement. This procedure maximizes the number of informative sites to analyze. The number of lines resampled for each population was chosen depending on the number of lines sequenced and the quality of those sequences (Supplementary Table S1). Positions and genes without valid information for at least this defined number of lines were discarded for the analysis. The ancestral state of each polymorphic site was inferred from the comparison with the outgroup species *D. simulans*. The genome reference sequence and annotations correspond to the 5.57 FlyBase release (20). Gene-associated recombination rate for 100 kb non-overlapping windows were retrieved from Comeron *et al.* (21).

#### Human population genomic data

Genome variation data and ancestral state of variants generated by the 1000GP Phase III (19), together with divergence estimates between humans and chimpanzees, were retrieved from PopHuman (15) in Variant Call Format (VCF). The dataset included 84.4 million variants detected across 2504 individuals from 26 different populations, which were mapped to the human reference genome version GRCh37/hg19. Reportedly inbred individuals (22) and non-accessible nucleotides (19) were discarded following the PopHuman methodology (15). Genome annotations were retrieved from GENCODE (release 27). Recombination rate values associated with each protein-coding gene were obtained from Bhérer (23) and correspond to the sex-average estimates.

### Estimation of the number of synonymous and nonsynonymous changes

Inferring the action of natural selection on coding sequences relies on the computation of polymorphism and divergence data on two distinct types of sites in the genome: one putatively selected (usually non-synonymous coding sites), and one putatively neutral (usually synonymous coding sites) (2). This implies assigning a selective class for each nucleotide site in the genome. This task is not trivial when different transcripts overlap a genomic region. For example, one nucleotide site can be a non-synonymous site for one transcript but a synonymous site for another nested gene transcript. In these cases, we assign the most selective constrained class to the nucleotide site. In the example, the site is considered non-synonymous.

### Exclusion of low-frequency variants

Slightly deleterious variants are mainly segregating at low frequency (8,24,25). These rare polymorphisms can be excluded from the analyses by specifying one or several threshold frequency values depending on the _FWW_MKT, the eMKT or the aMKT method. In addition, the aMKT allows removing high-frequency variants that might be due to polarization errors (10,13).

### Statistical analysis

For analyses including several protein-coding genes, users are recommended to select the option *Concatenate genes*. In this case, iMKT analyzes the selective regimes for the whole gene set instead of for each gene separately and applies a statistical test of heterogeneity of the selection acting among the analyzed genes (Cochran-Mantel-Haenszel statistic). In addition, the iMKT web server allows performing statistical enrichment analyses to assess whether a group of genes is either enriched or depleted of positively selected genes when compared to the complete genome distribution or to a second group of genes submitted by the user. In this case, the user should choose also the option *Compare against whole-genome distribution* or *Compare against a second dataset*. A resampling 95% confidence interval (CI) is generated by estimating *α* with the chosen MKT test for 100 bootstrap replicates by sampling genes with replacement within each group. In the asymptotic MKT, 95% CI intervals around the *α* estimation are already provided in the output.

### Output

The output of iMKT is an extensive report displayed as an HTML page. It contains several sections, starting with a summary table with the input parameters, a table with descriptive statistics, and the standard MKT table. Finally, the tests selected by the user are displayed below.

## PRACTICAL GUIDE TO THE iMKT WEBSITE

The iMKT site allows performing four MK-derived tests as a web-based service. The website is divided into different sections, each of which allows performing different types of analyses.

### MKT analysis

This page allows performing diverse MK-derived tests and estimating different selective regimes in your own data. The input can either be polymorphism and divergence data in two separate files, as described in the Methods section, or protein-coding sequences as aligned multi-FASTA files. When a multi-FASTA file is uploaded, the server outputs the DAF spectrum and the divergence calculations, which can be downloaded by the user and used in subsequent analyses. Note, however, that the former input type gives more flexibility to analyze any sort of functional site. As an example, you might want to test for selection at nonsynonymous coding sites (N) compared to synonymous coding sites (S) as the classical MKT was formulated, or to test for selection at Conserved Noncoding Sequences (CNS, N) compared to non-CNS (S) (26), etc. The choices are unlimited according to the user's needs.

### PopFly/PopHuman data analysis

If you want to analyze *D. melanogaster* or human protein-coding data, iMKT contains readily available variation data obtained from the largest genome variation datasets in each species (see *Methods*). The first step is to select the genes to be analyzed in the table displaying all the available genes. Genes are identified by either the Gene symbol or the FlyBase/Ensembl ID. Genes in the table can be sorted/filtered by chromosome and recombination rate, in addition to the Gene symbol and Flybase/Ensembl ID. In case the user needs to analyze a specific list of genes that cannot be easily filtered from the provided table (e.g. genes related to a specific pathway, as obtained from a search in KEGG (27), a list with those genes, were genes are identified by symbol or FlyBase/Ensembl ID, can be uploaded. Second, one or more populations on which to perform the analysis need to be specified. Third, one or more MK-derived methods can be chosen. Finally, advanced options are available to analyze all the genes as a group instead of analyzing them separately (option *Concatenate genes*), and to compare the results of this gene set against all the genes of the genome (option *Compare against whole-genome distribution*) or against a second group of genes provided by the user (option *Compare against a second dataset*). Potential applications include analyzing a single protein-coding gene or exploring different selective regimes in genes that are expressed tissues, anatomic structures, or developmental stages (28)).

### Other sections of the website

The iMKT website includes extensive methodological and technical documentation (see the section *Documentation* in the website), as well as a complete tutorial on the usage of iMKT, with step-by-step examples (see the section *Help and tutorial* from the main page). The website also contains sample files for each available type of analysis and links to related resources such as PopFly, PopHuman, and the iMKT R package.

## APPLICATION EXAMPLE OF THE iMKT WEBSITE

**Figure 2.**
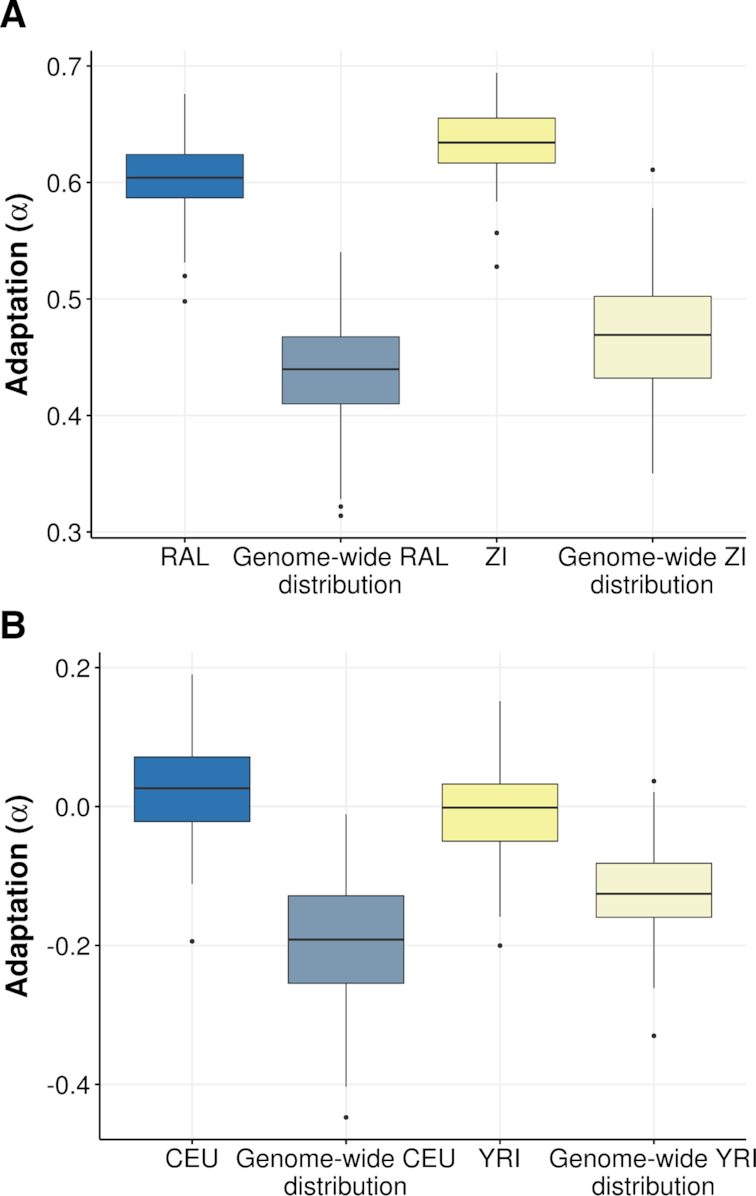
iMKT graphical output of an application example. Sampling distribution of *α* values for protein-coding genes located in regions of high recombination (recombination rate >7 cM/Mb) compared to all protein-coding genes in the genome for (**A**) the *D. melanogaster* Raleigh (RAL) population (blue) and the *D. melanogaster* Zambia (ZI) population (yellow) and (**B**) the human Utah Residents (CEU) population (blue) and the human Yoruba (YRI) population (yellow). The distribution was calculated by randomly sampling 400 genes 100 times from the two lists of genes with replacement and estimating *α* in each bin. Polymorphisms with a frequency below 0.05 in the analyzed population were discarded (see main text).

The iMKT website is designed to help testing evolutionary hypotheses from a population genetics perspective. The online tutorial, apart from guiding you in the usage of this resource, contains some worked-out cases that can be addressed using iMKT. In the application example developed here, we want to assess whether recombination rate limits the adaptive potential of protein-coding genes. The specific hypothesis is that genes located in high recombination regions undergo higher rates of adaptation. To test the hypothesis, we start by entering the *PopFly data analysis* page of iMKT. Next, we use the filtering options below the table to select 475 genes having a recombination rate higher than 7 cM/Mb (*Min recombination rate: 7*). After selecting the genes, we select one or more populations (*United States (RAL)*) and an MKT test (*eMKT*). Finally, we choose the option *Compare against whole-genome distribution*, which compares the distribution of *α* for the selected 475 genes located in regions of high recombination against the corresponding distribution for all *D. melanogaster* genes. As part of an extensive output report, an illustrative box plot shows a pronounced difference in the level of adaptation (*α*) between genes located in regions of high recombination (blue; *α* mean = 0.602; ±SD = 0.032) and all 13,753 *D. melanogaster* genes (orange; m *α* mean = 0.44; ±SD = 0.055) (Figure 2A).

We can repeat the same procedure for the *D. melanogaster* ancestral population from Zambia (*Zambia (ZI)*). As previously, the output report uncovers a much higher level of adaptation (*α*) in genes located in regions of high recombination (blue; *α* mean = 0.633; ±SD = 0.028) compared to the total 13 753 *D. melanogaster* genes (orange; *α* mean = 0.457; ±SD = 0.053) (Figure 2A).

Finally, the same analysis in humans for a colonizing population (*Utah Residents (CEU)*) and an ancestral population (*Yoruba (YRI)*) reveals negative *α* adaptation values in most cases and differences between the two groups of genes compared (Figure 2B). The results of this straightforward analysis show that: (i) *D. melanogaster* undergoes higher rates of adaptation than humans and (ii) genes located in regions of high recombination undergo higher rates of adaptation in both *D. melanogaster* and humans.

The example application developed here illustrates the power of iMKT to reveal new knowledge about evolutionary processes in Drosophila and humans without the need for labor-intensive data retrieval and/or processing by the user. The wide range of potential queries that can be performed using the searching capabilities of the iMKT website remarkably facilitates comprehensive analyses of evolutionary adaptation and constraint, even for non-bioinformaticians. As such, iMKT is a comprehensive reference site for the study of protein adaptation in massive population genomics datasets, especially in Drosophila and humans. Finally, we want to emphasize that the flexibility of iMKT to input custom data allows analyzing diversity data outside protein-coding regions. This expands, even more, the hypotheses that can be tested and makes iMKT a key tool to test for recurrent adaptation in the genome of any species.

## DATA AVAILABILITY

iMKT is a free resource online, open to all users without login requirement at https://imkt.uab.cat. The corresponding R package is available for download at https://github.com/BGD-UAB/iMKT.

## Supplementary Material

gkz372_Supplemental_File

## APPENDIX 1

### McDonald and Kreitman test (MKT)

The standard McDonald and Kreitman test (MKT) (2) was developed to be applied to protein-coding sequences, combining both divergence (*D*) and polymorphism (*P*) sites, and categorizing mutations as synonymous (*P*_S_, *D*_S_) and non-synonymous (*P*_N_, *D*_N_). If all mutations are either strongly deleterious or neutral, then *D*_N_/*D*_S_ is expected to roughly equal *P*_N_/*P*_S_. In contrast, if positive selection is operating in the region, adaptive mutations rapidly reach fixation and thus contribute relatively more to divergence than to polymorphism when compared to neutral mutations, and then *D*_N_/*D*_S_ > *P*_N_/*P*_S_ (Figure 1A)_._ Assuming that adaptive mutations contribute little to polymorphism but substantially to divergence, the proportion of non-synonymous substitutions that have been fixed by positive selection can be inferred as *α* = 1 – (*P*_N_/*P*_S_ × *D*_N_/*D*_S_) (7) (Figure 1B). The main limitation of the test is the presence in the population of non-synonymous slightly deleterious variants, biasing downward the estimates of adaptive evolution (*α*). Below are three proposed methods to correct the bias.

### Fay, Wyckoff and Wu correction (_FWW_MKT)

Because slightly deleterious variants tend to segregate at lower frequencies than do neutral mutations, Fay, Wyckoff and Wu or FWW correction (Figure 1C) propose to remove low-frequency polymorphisms from the analysis (8). *α* is estimated using the standard MKT equation but considering only those polymorphic sites (for both neutral and selected classes) with a frequency above an established cutoff. Charlesworth and Eyre-Walker (25) showed that even removing low-frequency variants, the estimate of *α* is still downwardly biased. Only these estimates are reasonably accurate when the rate of adaptive evolution is high and the distribution of fitness effects of slightly deleterious mutations is leptokurtic (because leptokurtic distributions have a smaller proportion of polymorphisms that are slightly deleterious).

### Extended MKT (eMKT)

Mackay *et al.* (9) proposed the extended MKT (Figure 1D). Instead of simply removing low-frequency polymorphism below a given threshold, the count of segregating sites in non-synonymous sites is partitioned in the number of neutral variants (using neutral sites as a proxy) and the number of weakly deleterious variants. This increases the power of detecting adaptive selection (as it does not remove as much data as the _FWW_MKT) and allows the independent estimation of both adaptive and weakly deleterious substitutions. *P*_N_, the count of segregating sites in the non-synonymous class, is discomposed into the number of neutral variants and the number of weakly deleterious variants, *P*_N_ = *P*_N neutral_ + *P*_N weakly del._ (9). The estimation of both numbers allows estimating positive (adaptive) and negative selection independently. *α* is estimated from the standard MKT table discounting weakly deleterious variants: *P*_N_ is substituted by the expected number of neutral segregating sites, *P*_N neutral_. The correct estimate of *α* is then *α* = 1 – (*P*_N neutral_/*P*_S_ × *D*_N_/*D*_S_).

### Asymptotic MKT (aMKT)

Messer and Petrov (10) proposed an asymptotic extension of MKT that takes slightly deleterious mutations into account and yields accurate estimates of *α* (Figure 1E). This method, named asymptotic MKT, is robust to the presence of selective sweeps (hitchhiking) and to the segregation of slightly deleterious substitutions (BGS). In this method, *α* is estimated in different frequency intervals (*x*) and these values are then adjusted to an exponential function, of the form: *α*_fit(*x*)_ = *a* + *b*^−*cx*^. The asymptotic *α* estimate is obtained by extrapolating the value of this function to *x* = 1: *α*_asymptotic_ = *α*_fit (*x* = 1)_.

The asymptotic MKT has been extended to estimate both positive (adaptive) and negative selection (Coronado-Zamora, submitted). aMKT requires a high volume of polymorphic data to fit the asymptotic function, being a suitable method in the case of concatenating numerous variants of multiple genes.
