# Supplementary material for: iMKT: the integrative McDonald and Kreitman test
Source: Nucleic Acids Res. 2019 May 13;47(W1):W283–8. doi: 10.1093/nar/gkz372 (PMC6602517; doi:10.1093/nar/gkz372)
Supplement: gkz372_Supplemental_File [file gkz372_supplemental_file.pdf]

Supplementary material

iMKT: the integrative McDonald and Kreitman test

Jesús Murga-Moreno, Marta Coronado-Zamora, Sergi Hervás, Sònia Casillas and Antonio Barbadilla*

Institut de Biotecnologia i de Biomedicina and Departament de Genètica i de Microbiologia, Universitat Autònoma de Barcelona, 08193 Bellaterra, Barcelona, Spain.

*To whom correspondence should be addressed. Tel: +34 93 5868941; Fax: +34 93 5812011; Email: antonio.barbadilla@uab.cat

Supplementary Table 1. Number of lines resampled in each *Drosophila* population available at iMKT.

**Population**	**Number of resampled lines**
RAL	160
USI	15
USW	27
CO	9
EA	10
EF	25
EG	10
GA	7
RG	21
SP	20
SD	30
ZI	154
CHB	12
FR	70
NTH	15
AUS	14
